# Associations between walking limitations and reported activity destinations among older adults

**DOI:** 10.1007/s10433-024-00813-1

**Published:** 2024-05-22

**Authors:** Essi-Mari Tuomola, Kirsi E. Keskinen, Taina Rantanen, Erja Portegijs

**Affiliations:** 1 Faculty of Sport and Health Sciences and Gerontology Research Center, University of Jyväskylä, P.O. Box 35 (viv), 40014 Jyvaskyla, Finland; 2 Center for Human Movement Sciences, University of Groningen, University Medical Center Groningen, Groningen, The Netherlands

**Keywords:** Mobility limitation, Activity destination, Aging, Participation, Built environment, Spatial mobility

## Abstract

In old age, walking difficulty may reduce opportunities to reach valued activity destinations. Walking modifications, e.g., slower pace or using a walking aid, may enable individuals to continue going where they wish, and hence postpone the consequences of the onset of walking difficulties. We studied visited activity destinations (type, distance) among older people with varying degrees of walking limitations. Community-dwelling 75–85-year-old people living in Jyväskylä (*N* = 901) were asked to state whether they had no difficulty walking 2 km, had modified their walking, or had difficulty walking. On a digital map, participants located physical exercise, attractive, and regular destinations they had visited during the past month. Destination counts and median distance to destinations from home were computed. Participants with intact walking reported higher counts of physical exercise (IRR = 1.45, 95% CI [1.31, 1.61]) and attractive destinations (IRR = 1.23, 95% CI [1.10, 1.40]) than those with walking difficulty and also visited these destinations further away from home than the others (*b* = 0.46, 95% CI [0.20, 0.71]). Those with walking modifications reported higher counts of physical exercise destinations than those with walking difficulty (IRR = 1.23, 95% CI [1.09, 1.40]). Counts of regular destinations and distance traveled were not associated with walking limitations. Walking modifications may help people with walking difficulty reach destinations further away from home, potentially contributing to their sense of autonomy. For those with walking difficulty, a low count of destinations other than regular destinations, e.g., shops or healthcare facilities, may signal their abandonment of recreational activities and a decrease in their life space, potentially leading to reduced well-being.

## Introduction

Mobility outside the home is important for healthy aging and the maintenance of older adults’ independence (Satariano et al. 2012). Mobility refers to the ability to move within one’s community environments either independently or by using assistive devices or vehicles (Webber et al. 2010). The most common reasons for older people making regular trips outdoors are running daily errands, shopping, walking, and meeting other people (Davis et al. 2011; Tsai et al. 2016; Chudyk et al. 2015). Visiting different destinations may increase daily physical activity (Tsai et al. 2016; Portegijs et al. 2015), maintain functional capacity and mobility, and enhance quality of life among older adults (Satariano et al. 2012).

The socio-ecological model posits that individual, social, and environmental factors influence older people’s possibilities to be active outside the home (Sallis et al. 2006; Chudyk et al. 2015). Functional decline may increase older people’s risk of developing walking difficulties and hence reduce their possibilities to participate in activities outside the home (Verbrugge and Jette 1994; Hoenig et al. 2006; Freedman et al. 2017; Rantakokko et al. 2009; Leppä et al. 2021) and carry out essential activities of daily living (Sugiyama et al. 2018). The most common reason preventing older people from engaging in outdoor activities is difficulty in walking (Wilkie et al. 2007). Walking limitations have been associated with decreased participation in leisure activities outside the home (Hand and Howrey 2019; Tuomola et al. 2023). Decreasing walking abilities also render older adults more vulnerable to environmental factors (Portegijs et al. 2017).

According to the ecological model of aging, walking abilities can be maintained by reducing task demands, increasing personal capacity, or lowering environmental demands (Lawton and Nahemow 1973). Older adults may modify their walking behavior when environmental demands increase relative to their physiological capacity (Freedman et al. 2017; Skantz et al. 2020a, b). The selective optimization with compensation (SOC) model proposed by Baltes and Baltes (1990) takes a similar approach. According to the model, older people need to select goals, optimize their resources to achieve those goals, and compensate for their reduced abilities to maintain functioning (Baltes and Baltes 1990). When older adults start experiencing a decline in their walking stamina, they may optimize their mobility by modifying their way of walking (Saajanaho et al. 2015; Siltanen et al. 2020). Such modifications, including walking at a slower pace, resting in the middle of walking, or using assistive devices, may help individuals continue walking to important destinations, at least in the earlier phases of physical capacity decline (Rantakokko et al. 2016; Skantz, Rantanen, Palmberg, et al. 2020). Thus, adaptive walking modifications often are the first signs of functional decline or preclinical disability (Fried et al. 2000).

The neighborhood environment may offer multiple destinations, such as shops and other commercial destinations, parks and other public open spaces, and recreational facilities that support older adults’ outdoor mobility (Sugiyama et al. 2012; Barnett et al. 2017). Such destinations provide opportunities for older people to be both physically active and interact with other people (Van Cauwenberg et al. 2018; Chaudhury et al. 2016; Nathan et al. 2012). Several studies have found that having walkable destinations in their neighborhood not only motivates older adults to walk (Nathan et al 2012; Gauvin et al. 2012; Barnett et al. 2017) and be physically active (King 2008; Barnett et al. 2017), but also slows down the development of walking difficulties (Eronen et al. 2013; Sugiyama et al. 2018). It is noteworthy that the type of destination may also influence the distance individuals are ready to travel (McCormack et al. 2006).

Online participatory mapping methods, such as the Public Participation Geographic Information System (PPGIS), provide an affordable and user-friendly way to examine the relationship between individuals and their environment (Laatikainen et al. 2018). The PPGIS allows researchers to collect information from a large group of individuals while minimizing the burden on participants (Hasanzadeh et al. 2017; Laatikainen et al. 2018; Portegijs et al. 2020; Schmidt et al. 2018). Previous studies have shown that PPGIS can achieve reasonable spatial accuracy when mapping physical features of the environment (Brown and Kyttä 2014) and validity in measuring frequently visited destinations and distance-related features (Hinrichs et al. 2020; Shareck et al. 2013). Locating destinations on a map has shown acceptable usability among older adults (Gottwald et al. 2016). Map-based questionnaires can provide information on people’s destinations and the locations in which they move (Kestens et al. 2017) and on their motives to visit specific destinations (Portegijs et al. 2021). Self-reports can yield information about personally meaningful environmental features (Portegijs et al. 2020). Map-based questionnaires allow investigation of older adults’ spatial behavior (Laatikainen et al. 2018) and the precise distances to their activity destinations (Portegijs et al. 2020).

We know relatively little about activity destinations that support older adults’ activity behavior outside the home, especially those of older adults with different kinds of walking limitations. The purpose of this study was to gain an understanding of how people in the earlier (preclinical walking modifications) and later (manifest difficulty walking) phases in the walking disablement process are reporting different activity destinations compared to those with intact walking ability. Hence, this study explored the associations of older adults’ walking limitations with destination counts and distances to activity destinations. Data on activity destinations were obtained with the PPGIS questionnaire and included regular destinations, physical exercise destinations, and attractive destinations.

## Methods

This study forms part of the Places of Active Aging project which links participant data on the “Active aging—resilience and external support as modifiers of the disablement outcome” (AGNES) study with map-based data. As described previously, the AGNES baseline data were collected during 2017–2018 (Rantanen et al. 2018). A random sample of community-dwelling 75-, 80-, and 85-year-old adults living in the city of Jyväskylä in Central Finland was drawn from the Digital and Population Data Services Agency in Finland (Rantanen et al. 2018). The inclusion criteria for the study were living in the study area and being community-dwelling, willingness to participate, and the ability to communicate and provide an informed consent. All participants lived in Jyväskylä, a medium sized city with 141 305 inhabitants (Official Statistics of Finland 2023). Our study area has small hills and quiet residential streets, with some busier streets intersecting them. City and subcenters form the service and residential areas and most of the shops and other services are concentrated in the city center. A total of 1 018 respondents participated in structured home interviews (Rantanen et al. 2018), of whom 908 participated in physical assessments in the research center, including a map-based assessment of their perceived environment. Of the participants in the map-based assessments, 901 located their activity destinations on a digital map with the assistance of an interviewer (Portegijs et al. 2021). Participants’ home addresses were also located on a map using the Digiroad dataset (Finnish Transport Infrastructure Agency 2019) in Geographic Information System (GIS) software ArcMap 10.6.1 (Esri Inc.). Participants had better health and mobility than nonparticipants (Portegijs et al. 2019). The study was conducted in accordance with the Declaration of Helsinki. The Ethical Committee of the Central Finland Health Care District approved the study. All participants gave their written informed consent at the start of the home interview.

### Main variables

*Walking limitations* were assessed based on self-reported walking difficulties and walking modifications. In the in-person interview, participants were asked the question “Do you have difficulty walking 2 km?”. The response categories were (1) able without difficulty, (2) able with some difficulty, (3) able with a great deal of difficulty, (4) unable without the help of another person, and (5) unable to manage even with help. To identify participants using walking modifications, participants who reported being able to walk two kilometers without difficulty (response category 1) were asked an additional question: “Have you noticed any of the following changes when walking two kilometers due to your health or physical functioning?” The walking modifications listed were walking slower, using an aid, resting during walking, reduced the frequency of walking, and given up walking distances of two km. For each modification option, participants indicated whether they were using that modification (yes/no). For the analyses, participants were categorized into three groups: (a) intact walking (reporting neither difficulty nor modifications), (b) walking modifications (reporting no difficulties and ≥ 1 modification), and (c) walking difficulty (reporting at least some difficulty).

A map-based internet questionnaire on *activity destinations* was administered using the interactive online Maptionnaire® tool (Mapita LTD). Participants were asked to locate on a map three types of activity destinations which they had visited several times during the past month. These predefined activity destination types were 1) destinations for physical exercise, 2) destinations regarded as attractive for other out-of-home activity, and 3) destinations for regular activities (not related to physical exercise). Physical exercise destinations included outdoor and indoor sports facilities and outdoor recreational areas. Attractive destinations included destinations which served as motivators for older people to engage in out-of-home activities (other than physical exercise), such as nature settings, lakeside areas, services, and events, places to rest and other infrastructure-related places. Regular destinations included essential destinations, e.g., grocery stores and other shops, food and health services, and destinations for self-selected activities such as organized activities and social visits.

To reflect diversity in destinations for each participant, the reported number of physical exercise, attractive, and regular destinations was counted for each respective category and summed to yield a total count of activity destinations. Distances between participants’ homes and their reported destinations were computed as road network distances (expressed in meters) using the Digiroad dataset (Finnish Transport Infrastructure Agency 2019). For technical reasons, distances to 19 destinations (e.g., an island or abroad) were defined manually using Google Maps. The median distance was calculated for all reported activity destinations combined as well as separately for each activity destination type.

### Covariates

Age, sex, perceived financial situation, years of education, cognitive function, regular driving, and residential density were used as covariates in the analyses based on existing knowledge of variables that correlate with out-of-home mobility. Participants’ age and sex were drawn from the Digital and Population Data Services Agency in the context of their recruitment. Perceived financial situation and years of education, which were used as indicators of socioeconomic status, were obtained during the home interview. Participants were asked to rate their perceived financial situation on a four-point scale ranging from very good to poor, and responses were recoded as “good to very good” versus “poor to fair.” Educational level was self-reported as years of full-time education. Cognitive function was measured using the Mini-Mental State Examination during the home interview (MMSE; Folstein et al. 1975). The MMSE score ranges from 0 to 30, with a higher score indicating better function. Regular driving was assessed with the question “How often do you drive a car yourself?” For the analyses, driving a car was divided into two groups: driving regularly (daily or weekly) versus driving rarely (monthly or less frequently). Residential density was used as an indicator of the availability of services and the amount of infrastructure for outdoor mobility. The range of residential density in the 1 km × 1 km squares in the study area (Population Grid Data 2018) was categorized in tertiles (lowest, middle, highest). Each participant was assigned to the population density tertile of their home location.

### Statistical analyses

Descriptive statistics by the walking limitation categories were reported in percentages for categorical variables and as medians with interquartile range (IQR) for continuous variables. Differences between groups were tested with a Chi-square test or the Kruskal–Wallis test. The associations between reported walking limitations and counts of activity destinations were assessed cross-sectionally using Poisson loglinear regression analysis. General linear model analyses were used to investigate the associations between walking limitations and the log-transformed median distance from home to the reported activity destination. In all analyses, those with walking difficulty were used as a reference group. Analyses were run separately for each activity destination type and for all destinations combined. The Poisson loglinear regression models were adjusted for age, sex, perceived financial situation, years of education, MMSE score, regular driving, and residential density. General linear models were first adjusted for age and sex and then for age, sex, perceived financial situation, years of education, MMSE score, regular driving, and residential density.

Of the 901 participants, 14 were excluded from the analysis due to missing information on self-reported walking limitations, and hence, the analysis was conducted for 887 participants. Information was missing on years of education for four participants, MMSE score for three participants, and financial situation for four participants. These 11 participants were not included in the fully adjusted models in the Poisson loglinear regression and general linear model analyses. We did additional sensitivity analyses stratifying the data based on regular driving. In stratified analyses, the main models did not materially differ between drivers and non-drivers (data not shown). The results were regarded as statistically significant if the *p* value was < 0.05 or 95% confidence intervals did not include one in the Poisson loglinear regression analyses or did not include zero in the general linear model analyses. SPSS Statistics for Windows (version 26.0; IBM Corp.) were used for statistical analyses.

## Results

The participants’ median age was 78.9 (IQR = 4.7) years and 57.1% (*n* = 506) of the participants were women. Participants with intact walking were statistically significantly more often male, younger, drove regularly and had a higher education, better financial situation, and higher MMSE score than those with walking difficulties (*p* ≤ 0.002 for all; Table 1).

**Table 1:** Participants characteristics by walking limitations (*N* = 887)

	Intact walking *n* = 424	Walking modifications *n* = 167	Walking difficulty *n* = 296	*p* value
	Median (IQR)	Median (IQR)	Median (IQR)	
Age, years	75.7 (4.4)	79.4 (4.6)	79.6 (8.6)	** < 0.001**^**a**^
Education, years	11.0 (6.0)	10.0 (7.0)	10.0 (6.0)	**0.002**^**a**^
MMSE, score	28.0 (2.0)	28.0 (3.0)	27.5 (3.0)	**0.002**^**a**^
Men, % (*n*)	49.1 (208)	43.1 (72)	34.1 (101)	**0.001**^**b**^
Good or very good perceived financial situation % (*n*)	69.5 (294)	54.5 (91)	51.5 (151)	** < 0.001**^**b**^
Regular driving % (*n*)	64.2 (272)	55.7 (93)	43.6 (129)	** < 0.001**^**b**^
Tertile of residential density % (*n*)				0.218^b^
Lowest	48.1 (204)	40.7 (68)	49.0 (145)	
Middle	20.0 (85)	28.1 (47)	20.6 (61)	
Highest	31.8 (135)	31.1 (52)	30.4 (90)	

Statistically significant *p* values are bolded. Bold values indicate *p* < 0.05. *IQR* interquartile range, *MMSE* Mini-Mental State Examination

^a^Tested with Kruskal–Wallis test. ^b^ Tested with Chi-square test

### Count of activity destinations

The most commonly reported physical exercise destinations were outdoor sports facilities, while the most reported attractive destinations were service or event venues and nature settings (Appendix 1). The most commonly reported regular destinations were grocery and other stores. The characteristics of the participants’ activity destinations by walking limitations are summarized in Table 2. The results showed that the median count of destinations reported by the participants with intact walking was seven, whereas the corresponding count reported by those with walking difficulty was six (*p* < 0.001; Table 2). In addition, compared to participants with walking difficulty, those with intact walking reported a higher count of physical exercise destinations (median = 3, IQR = 2 vs. median = 2, IQR = 2; *p* < 0.001) and attractive destinations (median = 2, IQR = 1 vs. median = 1, IQR = 2; *p* < 0.001). No statistically significant differences were observed in regular destination counts between the walking limitations categories (*p* = 0.410).

**Table 2:** Characteristics of reported activity destinations by walking limitations (*N* = 887)

	Intact walking *n* = 424	Walking modifications *n* = 167	Walking difficulty *n* = 296	*p* value
	Median (IQR)	Median (IQR)	Median (IQR)	
Count				
All destinations	7.0 (3.0)	6.0 (3.0)	6.0 (4.0)	** < 0.001**^a^
Physical exercise destinations	3.0 (2.0)	2.0 (2.0)	2.0 (2.0)	** < 0.001**^a^
Attractive destinations	2.0 (1.0)	1.0 (2.0)	1.0 (2.0)	**0.003**^a^
Regular destinations	2.0 (1.0)	3.0 (1.0)	2.0 (1.0)	0.410^a^
Median distance (km)				
All destinations	2.1 (1.7)	1.6 (1.6)	1.4 (1.6)	** < 0.001**^a^
Physical exercise destinations	1.9 (1.8)	1.3 (1.4)	0.9 (1.4)	**0.001**^a^
Attractive destinations	2.0 (10.4)	1.1 (3.7)	0.9 (2.9)	** < 0.001**^a^
Regular destinations	2.5 (2.8)	2.2 (2.5)	2.1 (2.4)	**0.048**^a^

Statistically significant *p* values are bolded. Bold values indicate *p* < 0.05. *IQR* interquartile range, *MMSE* Mini-Mental State Examination

^a^Tested with Kruskal–Wallis test. ^b^ Tested with Chi-square test

**Fig. 1:**
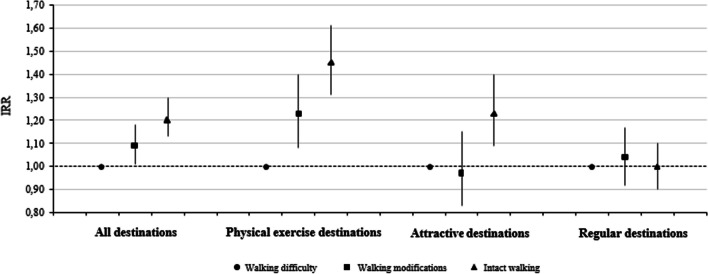
The incidence rate ratios (IRR) and 95% confidence intervals (CI) for the count of activity destinations in Poisson loglinear regression models with walking limitations (*N* = 887). The results were considered statistically significant when the 95% confidence intervals did not include one

Figure 1 presents the fully adjusted incidence rate ratios (IRR) and 95% confidence intervals (CI) for the activity destination counts for those with walking limitations. Compared to the participants with walking difficulty, the IRR for the count of all destinations combined for those with intact walking was 1.20 (95% CI [1.13, 1.28]). Thus, the total count of destinations reported by those with intact walking was 21% higher than that reported by those with walking difficulty. Intact walkers had greater IRRs for the counts of physical exercise destinations (IRR = 1.45, 95% CI [1.31, 1.61]) and attractive destinations (IRR = 1.23, 95% CI [1.09, 1.40]) than those with walking difficulty. Participants with walking modifications were estimated to report a 9% higher count of all destinations combined (IRR = 1.09, 95% CI [1.01, 1.18]) and a 23% higher count of physical exercise destinations (IRR = 1.23, 95% CI [1.08, 1.40]) than those with walking difficulty. However, the association between using walking modifications and reporting attractive destinations was nonsignificant. The results also showed that having walking limitations was not statistically significantly associated with the reported count of regular destinations.

### Median distance to activity destinations

Table 2 reveals that, when all destinations were considered, older people with intact walking reported destinations approximately 700 m further from their homes than those with walking difficulty (*p* < 0.001). The physical exercise destinations reported by intact walkers were located one kilometer (*p* = 0.001) and the attractive destinations 1.1 km (*p* < 0.001) further than those reported by those with walking difficulty. The difference in the median distance of regular destinations between participants with intact walking and those with walking difficulty was 400 m (*p* = 0.048). In general, the linear models using loglinear transformation showed that the older people with intact walking reported a greater median distance to all destinations combined (*b* = 0.13, 95% (CI) [0.08, 0.19]), physical exercise destinations (*b* = 0.61, 95% CI [0.47, 0.74]), and attractive destinations (*b* = 0.51, 95% CI [0.26, 0.77]) than those with walking difficulty (Table 3). After adjusting with covariates, the associations were somewhat attenuated but remained significant in all models. In addition, participants using walking modifications (*b* = 0.42, 95% CI [0.25, 0.59]) reported a greater median distance to physical exercise destinations than participants with walking difficulty. The association remained statistically significant after adjusting with covariates. However, the association between walking limitations and median distance to regular destinations was nonsignificant.

**Table 3:** General linear model analyses of the associations between walking limitations and median distance to reported activity destinations (*N* = 887)

	Distance to all destinations				Distance to physical exercise destinations				Distance to attractive destinations				Distance to regular destinations			
	Crude^a^		Fully adjusted^b^		Crude^a^		Fully adjusted^b^		Crude^a^		Fully adjusted^b^		Crude^a^		Fully adjusted^b^	
	b	95% CI	b	95% CI	b	95% CI	b	95% CI	b	95% CI	b	95% CI	b	95% CI	b	95% CI
Intact walking (vs. walking difficulty)	**0.13**	**0.08, 0.19**	**0.13**	**0.07, 0.19**	**0.61**	**0.47, 0.74**	**0.58**	**0.44, 0.71**	**0.51**	**0.26, 0.77**	**0.46**	**0.20, 0.71**	0.05	−0.05, 0.14	0.04	−0.05, 0.13
Walking modifications (vs. walking difficulty)	0.05	−0.02, 0.12	0.05	−0.01, 0.12	**0.42**	**0.25, 0.59**	**0.41**	**0.25, 0.58**	0.05	−0.26, 0.36	−0.10	−0.33, 0.30	0.04	−0.08, 0.15	0.05	−0.06, 0.16
Men (vs. women)	**0.17**	**0.12, 0.22**	**0.10**	**0.42, 0.15**	0.01	−0.10, −0.13	−0.00	−0.14, 0.71	0.04	−0.18, 0.26	0.10	−0.15, 0.36	**0.20**	**0.12, 0.28**	0.01	−0.02, 0.13
Good or very good perceived financial situation (vs. poor to fair)			0.01	−0.04, 0.07			0.04	−0.08, 0.17			0.05	−0.16, 0.30			0.03	−0.06, 0.11
Age	**−0.02**	**−0.03, −0.02**	**−0.02**	**−0.03, −0.01**	**−0.02**	**−0.04, −0.01**	**−0.02**	**−0.04, 0.00**	−0.02	−0.05, 0.01	−0.02	−0.05, 0.01	**−0.01**	**−0.02, −0.00**	−0.01	−0.02, 0.00
Years of education			−0.01	−0.01, 0.00			0.00	−0.01, 0.02			−0.00	−0.03, 0.03			**−0.02**	**−0.03, −0.00**
MMSE score			−0.01	−0.01, 0.02			0.03	−0.01, 0.05			0.02	−0.03, 0.07			0.00	−0.00, 0.03
No regular driving (vs. regular driving)			**−0.12**	**−0.12, −0.06**			−0.06	−0.20, 0.08			0.03	−0.19, 0.28			**−0.23**	**−0.32, −0.13**
Lowest tertile of residential density (vs. highest tertile)			**0.17**	**0.12, 0.23**			0.08	−0.06, 0.22			**−0.33**	**−0.59, −0.07**			**0.23**	**0.14, 0.32**
Middle tertile of residential density (vs. highest tertile)			**0.15**	**0.08, 0.21**			0.08	−0.09, 0.24			−0.12	−0.42, 0.19			0.05	−0.06, 0.16

*b* Regression coefficient, *CI* confidence interval, *MMSE* Mini-Mental State Examination. General linear models adjusted for: ^a^age and sex; ^b^age, sex, perceived financial situation, years of education, MMSE score, regular driving, and residential density. Values in bold; if the 95% CI does not contain the value 0, *p* < 0.05

## Discussion

In this study, use of a map-based PPGIS method enabled us to obtain new information about activity destination counts and locations relative to the homes of older adults. Compared to the participants with walking difficulty, the older adults with intact walking reported higher counts of physical exercise and attractive destinations and also destinations that were located further away from their homes. Those using walking modifications, indicative of early limitations, reported a higher count of physical exercise destinations than those with walking difficulty. However, the association between distance to destinations for regular activities and walking limitations was nonsignificant. As far as we know, this is the first study to examine the associations between manifest and early walking limitations and different types of activity destinations among older adults. The validity of these findings is supported by previous results, indicating that destinations may motivate older adults to participate in out-of-home activities and that older adults have multiple reasons for visiting places outside the home (Tsai et al. 2016; Chudyk et al. 2015). However, earlier studies have also shown that having walking limitations may restrict people’s participation in activities outside the home (Hand and Howrey 2019; Tuomola et al. 2023), as we also found in relation to the destinations visited by our participants.

The ecological model of aging suggests that when older people encounter environmental challenges that exceed their physical capabilities, they may adjust their walking behavior, e.g., by reducing their walking pace, using assistive devices, or taking breaks to reduce the physiological demands of walking (Freedman et al. 2017; Skantz et al. 2020a, b). This aligns with the model of selection, optimization, and compensation, which suggests that older individuals use these strategies to continue engaging in activities that are important to them (Baltes and Baltes 1990). Walking adaptations enable older adults to maintain a sufficient level of community mobility (Skantz, Rantanen, Palmberg, et al. 2020). Our results complement earlier findings by showing that walking modifications allow older people to continue visiting destinations where activities meaningful to them take place. In the current study, although the older adults who had modified their walking behavior reported more physical exercise destinations than those with walking difficulty, the two groups showed only a nonsignificant difference in counts of destinations regarded as attractive, including nature locations. A possible explanation might be that their neighborhood environments may lack the kinds of facilitators that support and motivate older people with walking limitations to visit such places. Older people with walking difficulties experience environmental features differently from intact walkers (Sakari et al. 2017; Skantz et al. 2020a, b). Lack of resting places, long distances to destinations, or hilly terrain may further encumber their mobility (Rantakokko et al. 2012; Keskinen et al. 2020).

In our study, the most commonly reported destinations that people regarded as regular were grocery and other stores. As daily routines, visits to regular destinations (Chudyk et al. 2015; Davis et al. 2011) may form a major part of people’s community mobility, especially for older people with reduced walking ability. This was also evident in our data. Older adults reported an equal count of regular destinations irrespective of the presence or absence of walking modifications or difficulty even though those with walking difficulty reported a lower count of other activity destinations. A low count of physical exercise and attractive destinations may signal a reduction in recreational activities, leading to decreased life-space and reduced well-being in old age.

Interestingly, regular destinations were located further away from home than physical exercise and attractive destinations. This is most likely because they were critical, such as grocery stores, health services, and other shops. It has previously been established that critical destinations may be located further away from home and still be visited regularly, although also using other modes of transport than walking. (Hirsch et al. 2016; Nathan et al. 2012). According to a previous study, passive modes of transportation such as cars or public transportation were commonly used for daily trips to services and shops (Sugiyama et al. 2019). Shopping trips, in particular, often involve carrying groceries and are more likely to be traveled by car. In our study, the median distance to regular destinations was from 2.1 to 2.5 km. This suggests that older adults with walking limitations travel outside their neighborhood to access services and shops, which contributes to their daily activity (Hillsdon et al. 2015) and well-being (Satariano et al. 2012). Our recent study indicated that almost all self-selected activities promote well-being (Rantanen et al. 2021). This study suggests that the environmental characteristics of the living environment, such as the residential density of the neighborhood, can influence how far older adults travel to visit different places. Urban areas may offer more destinations that are closer to home. Older adults may walk instead of driving if the meaningful destinations are located nearby (Rosso et al. 2013; Chudyk et al. 2015).

We also found associations between the extent of walking limitations and distances to specific destinations. Those with intact walking and those using modifications reported physical exercise destinations further from home than those with walking difficulty. This may suggest that they have more physical reserves and are thus willing to travel further to a specific type of destination. Previous studies have shown that mobility restrictions are associated with a smaller activity range (Iveson et al. 2023) and lower life-space mobility (Dunlap et al. 2022), indicating that older adults with mobility limitations may have more limited use of their environment (Iveson et al. 2023). The physical exercise and attractive destinations reported by those with walking difficulty were all within one kilometer from home. This underlines the importance of the local siting of services and other important destinations.

The strengths of this study include a population-based sample of individuals aged 75–85 who were interviewed face-to-face using an online participatory mapping method, the PPGIS, to study the out-of-home activity destinations of older adults. We studied road network distances to these locations rather than straight-line distances. This study provided a comprehensive picture of older adults’ activity destinations including not only destinations for daily errands but also those for physical exercise and enjoying outdoor mobility. In addition, our sample size was relatively large and missing data were few. However, the study has its limitations. The cross-sectional design limits the ability to infer causality. This study was also conducted in one country, Finland, and therefore generalization to different cultural and geographic contexts must be carefully considered. Moreover, our study population comprised relatively healthy and well-functioning older people. We cannot rule out variation in the accuracy of the identified locations, although previous research has shown that the spatial quality of the PPGIS may be adequate for mapping daily mobility (Laatikainen et al. 2018).

## Conclusions

Participants with intact walking reported more physical exercise destinations and attractive destinations than participants with walking difficulty. Moreover, intact walkers’ destinations were located further away from home. Walking modifications, such as resting and using a walking aid, may help individuals to continue visiting meaningful destinations, especially physical exercise destinations, despite functional decline. Our study suggests that despite the onset of walking difficulties, older people do not readily give up accessing destinations necessary for daily living. Such destinations may not only encourage older people to go outdoors but also give them an opportunity to be socially active. However, walking difficulties seem to decrease participation in recreational activities. Understanding the diversity of activity destinations and environments that are relevant for older adults without and with walking difficulties or early signs of walking limitations is important for designing age-friendly environments. These environments may encourage older people to be physically active despite early signs of walking difficulties. More research is needed on how environmental factors facilitate outdoor mobility and influence older people’s decisions about which destinations to visit. It would also be important to study the role of destinations in relation to overall physical activity and other health outcomes.

## Data Availability

Data will be made available on request. To request the data, please contact Professor Taina Rantanen (taina.rantanen@jyu.fi).

## Appendix 1: Percentages and counts of reported reasons to visit activity destinations by destination type

**Table:**

Physical exercise destinations	% ( *n* )
Outdoor sports facilities	58 (1350)
Indoor sports facilities	25 (575)
Outdoor recreational areas	17 (383)

Attractive destinations	% ( *n* )
Services and events	28 (595)
Nature	15 (326)
Appealing landscape	12 (254)
Waterbody or lake	11 (237)
Park or other green area	8 (162)
Good walkways or routes	7 (147)
Resting place	3 (57)
Even sidewalks	2 (49)
Other	14 (298)

Regular destinations	% ( *n* )
Grocery store	44 (1397)
Other store	16 (507)
Home of Friend/relative	9 (272)
Other service	6 (201)
Organized activity	6 (182)
Health service	5 (163)
Food service	4 (132)
Events	3 (104)
Church/parish	1 (37)
Cemetery	1 (32)
Other	5 (158)
